# Effects of Isothermal Temperature and Soaking Time on Water Quenched Microstructure of Nickel-Based Superalloy GH3536 Semi-Solid Billets

**DOI:** 10.3390/ma14164668

**Published:** 2021-08-19

**Authors:** Guanfei Xiao, Jufu Jiang, Ying Wang, Yingze Liu, Ying Zhang, Yinfeng Tian

**Affiliations:** 1 School of Materials Science and Engineering, Harbin Institute of Technology, Harbin 150001, China; guanfeixiao@163.com (G.X.); liuyingze1995@foxmail.com (Y.L.); 15165196982@163.com (Y.Z.); madfeng2021@163.com (Y.T.); 2 School of Mechatronics Engineering, Harbin Institute of Technology, Harbin 150001, China

**Keywords:** GH3536 alloy, semi-solid processing, microstructure, precipitate

## Abstract

Semi-solid billets of GH3536 alloy were prepared by semi-solid isothermal treatment of wrought superalloy method. GH3536 samples were soaked at several semi-solid temperatures (1350 °C, 1360 °C, 1364 °C, and 1367 °C) for 5–120 min. The effects of temperature and soaking time on the microstructure of GH3536 billets were studied. The results indicated that the microstructure was affected by coalescence mechanism, Ostwald ripening mechanism, and breaking up mechanism. Semi-solid microstructure of GH3536 alloy was composed of spherical solid particles and liquid phases, and the liquid phases affected the microstructure greatly. At 1350 °C, the coalescence mechanism was dominant at the early stage of isothermal treatment, then the Ostwald ripening mechanism played a major role for the longer soaking times. At higher temperatures, the breaking up mechanism occurred to form large irregular grains and small spherical grains. As the heating continued, the Ostwald ripening mechanism was dominant. However, at 1364 °C and 1367 °C, the solid grains had irregular shapes and large sizes when the isothermal time was 120 min. The optimum parameters for the preparation of GH3536 semi-solid billets were: temperature of 1364–1367 °C and soaking time of 60–90 min.

## 1. Introduction

Nickel-based superalloy is widely used for the manufacture of critical components of aero-engines owing to the excellent creep performance, corrosion resistance and abrasion resistance at high temperatures [1,2,3,4]. However, because of the poor hot working performance, nickel-based superalloy is difficult to form components through traditional forging techniques [5]. Meanwhile, the parts formed by casting methods have some defects such as segregation and porosity [6]. Therefore, it is necessary to pay more attention to the development of a new processing method on nickel-based superalloys.

Semi-solid processing (SSP) is a novel technology to form near-net shaped components of metal materials [7]. It combines both the advantages of casting and forging, and can form parts with good comprehensive performance and complex geometry. Recently, many aluminum and magnesium products have been fabricated by semi-solid forming [8,9,10,11], especially in the field of producing automotive parts. However, the SSP method of high melting point alloys has not received widespread attention because of the high semi-solid temperatures. The requirements (suitable semi-solid temperature range) of SSP for metallic materials are not high, so it is possible to adopt SSP on nickel-based superalloys. Besides, SSP also has many advantages, such as smooth filling, fewer defects and low deformation resistance. At the same time, SSP is easy to near-net forming, which can solve the problem of difficult cutting of nickel-base superalloys. Hence, it is meaningful to study the SSP of nickel-based superalloys.

SSP mainly includes two parts: the preparation of semi-solid billet (slurry) and subsequent forming, in which the key procedure is to fabricate semi-solid billet with globular microstructure [12]. In recent years, semi-solid billet preparation methods mainly include liquid-state method and solid-state method [13]. Solid state route does not deal with molten metal and reduces the pollution of semi-solid billets, and it has other advantages such as short process and low cost. In addition, because superalloys have high semi-solid temperatures, the solid-state method is more suitable to fabricate semi-solid billets. Strain induced melting activated (SIMA) [14,15,16] and recrystallization and partial melting (RAP) [17,18,19] are two common solid-state routes. However, both SIMA and RAP involve warm working or hot working before reheating. For the sake of shorten the procedures and decrease the cost, a new method named semi-solid isothermal treatment of wrought superalloy (SSITWS) [20] was developed to prepare semi-solid billets. The main factors affecting the microstructure of semi-solid billets are temperature and soaking time. Many researchers have investigated the impacts of these two factors on the microstructure of aluminum alloys [21,22], magnesium alloys [23,24], and steels [25,26]. However, the semi-solid characteristics of nickel-based superalloys has been rarely reported in literature at present.

The most remarkable feature of semi-solid billet is that its microstructure is a mixed microstructure with the coexistence of solid phase and liquid phase. During the heating process of semi-solid billet, when the heating temperature is higher than the solidus temperature, the incipient melting phenomenon will occur, resulting in the formation of liquid phase [27]. Mohamed at al. [28] pointed out that the incipient melting would occur on the grain boundaries of Al–Si–Cu–Mg cast alloys during semi-solid processing, leading to the formation of Al_2_Cu phase. Meng et al. [25] reported that due to the partial melting of austenite near the liquid phase and the dissolution of alloying elements in the liquid phase of SKD61 tool steel, the eutectic mixture formed from liquid phase after rapid cooling. The microstructure evolution of semi-solid billet mainly concerns the coarsening and spheroidization of solid grains, in which the coalescence mechanism and the Ostwald ripening mechanism are the main coarsening mechanisms. The coalescence mechanism [29] is caused by the combination of two or more adjacent grains with similar sizes. The Ostwald ripening mechanism [30] refers to the phenomenon that smaller grains dissolve and larger grains continue to grow, resulting in the increase of average grain size. In addition to the above two coarsening mechanisms, the breaking up mechanism will also appear in the microstructure evolution. Meng et al. [18] pointed out that due to the aggregation of intragranular droplets, large droplets near the grain boundaries would break the solid grains, resulting in the formation of small grains, thus reducing the average grain size.

In this work, aiming to develop a new forming technology of nickel-based superalloys, SSP of GH3536 alloy was investigated. GH3536 semi-solid billets were manufactured by SSITWS method. The effects of temperature and soaking time on the microstructure of GH3536 semi-solid billets were studied and discussed.

## 2. Materials and Methods

### 2.1. Materials

The as-received material in this study was a 38 mm diameter GH3536 extruded bar. The chemical composition (wt %) was 21.09 Cr, 17.91 Fe, 8.39 Mo, 0.77 Co, 0.15 Cu, 0.01 Nb, and a balance of Ni, which was determined by X-ray fluorescence. Differential scanning calorimetry (DSC) technique was conducted to obtain the solidification range of GH3536 alloy.

### 2.2. Preparation of Semi-Solid Billets

According to the SSITWS method [20], the extruded GH3536 specimens (10 × 10 × 8 mm^3^) were directly heated to semi-solid temperatures with the heating rate of 10 °C/min, then soaked for specified times. The experiments were conducted in a vacuum atmosphere furnace (ZY-QL1700, Luoyang Zhongyuan experimental electric furnace factory, Luoyang, Henan, China) with argon protection. The selected heating temperatures were 1350 °C, 1360 °C, 1364 °C, and 1367 °C. The soaking times were 5 min, 15 min, 30 min, 60 min, 90 min, and 120 min, respectively. When the heating process was finished, the GH3536 specimens were put into water for quenching immediately.

### 2.3. Observation of Microstructure

For microstructure observation, the quenched GH3536 specimens were grinded and polished first, then etched in a solution of 75 mL HCl and 25 mL HNO_3_ for 120 s. The optical microstructures of GH3536 specimens were observed by a Leica Dmi3000M optical microscope (OM) from the Leica Company in Munich, Germany. Zeiss scanning electron microscope (SEM) and energy dispersive spectrometer (EDS) from Leica Company in Munich, Germany were used to analyze the distribution of alloying elements. The microstructure characteristics were quantitatively evaluated by average grain size (D) and shape factor (F), and the calculation formulas were

(1) $$D=\frac{\sum_{i=1}^{N}\sqrt{4A_{i}/\pi }}{N}$$

(2) $$F=\frac{\sum_{i=1}^{N}4\pi A_{i}/P_{i}^{2}}{N}$$

where *A_i_* and *P_i_* were the area and the perimeter of the *i*th grain, respectively, and *N* presented the quantity of grains.

## 3. Results

### 3.1. As-Received GH3536 Alloy

**Figure 1:**
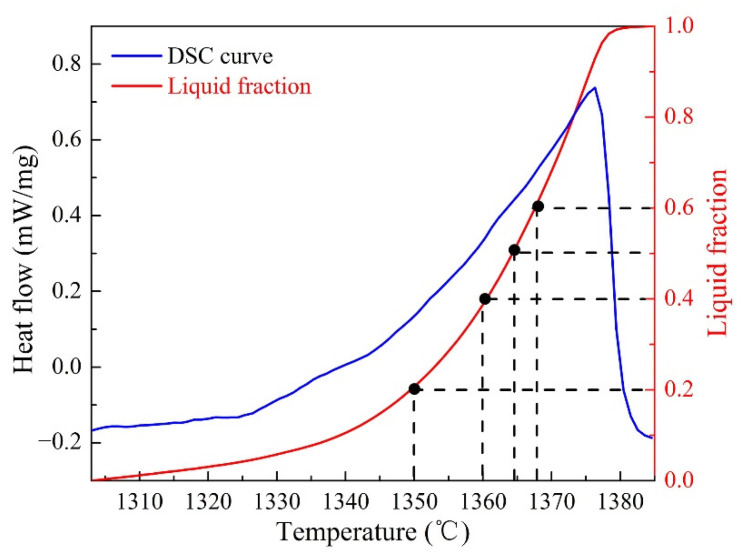
DSC curve and liquid fraction curve of GH3536 alloy.

The blue curve in Figure 1 presents the relationship between heat flow and temperature of the as-received GH3536 alloy, and the semi-solid interval of 1302–1385 °C were obtained. The DSC curve of GH3536 alloy at solid-liquid temperature range was integrated to obtain the total area, and then the liquid fraction was obtained by dividing the integral area of any temperature by the total area, as shown the red curve in Figure 1. According to Figure 1, when the semi-solid temperatures were 1350 °C, 1360 °C, 1364 °C, and 1367 °C, the corresponding liquid fractions were 20%, 40%, 50%, and 60%, respectively.

**Figure 2:**
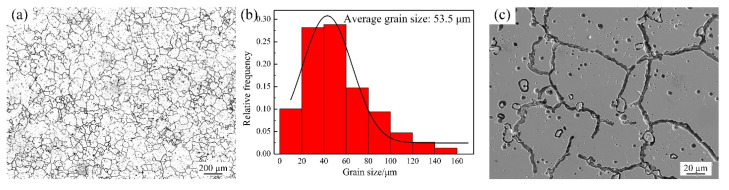
Microstructure of the as-received GH3536 alloy: (**a**) OM image, (**b**) histogram of grain size distribution, and (**c**) SEM image.

Figure 2a presents the OM image of the as-received GH3536 alloy, which exhibited an equiaxed grain structure with inhomogeneous grain sizes. Figure 2b is the histogram of grain size distribution, it can be found that the average grain size of the as-received samples was 53.5 ± 30.0 μm and its shape factor was 0.78 ± 0.12. Figure 2c illustrates the SEM microstructure of the as-received GH3536 alloy, it can be observed that the grains were roughly polygonal, and due to the difference of alloying elements between the grain boundaries and the grain interiors, the grain boundaries of the alloy presented obvious etching traces.

### 3.2. Microstructure Evolution of GH3536 Semi-Solid Billets

**Figure 3:**
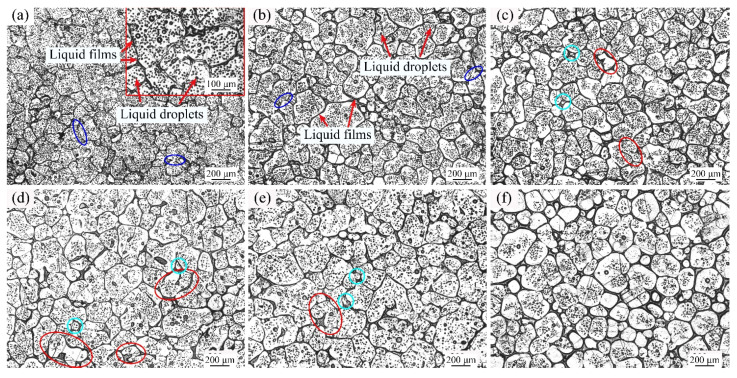
Optical micrographs of GH3536 alloy heat treated at 1350 °C for (**a**) 5 min, (**b**) 15 min, (**c**) 30 min, (**d**) 60 min, (**e**) 90 min, and (**f**) 120 min.

Figure 3 are the optical micrographs of GH3536 alloy heated at 1350 °C for various heating times. These microstructures all presented typical semi-solid characteristics, including solid grains, liquid droplets and liquid films. When GH3536 alloy was heated at 1350 °C for 5 min (Figure 3a), the solid grains with the average size of 122.7 μm had irregular shapes (shape factor of 0.78), and they were closely connected with each other. In Figure 3a, the liquid phase mainly consisted of many small intragranular liquid drops, while the proportion of liquid phase between the solid grains was relatively small. As shown in Figure 3b, the average size and shape factor of the solid grains increased to 157.4 μm and 0.81, respectively. At 30 min (Figure 3c), the average size (149.6 μm) and shape factor (0.81) of the solid grains were almost the same as those at 15 min. However, the distribution of liquid films on the grain boundaries increased. As shown in Figure 3d,e, the solid grains continued to grow, and their average grain sizes increased to 180.0 μm and 178.2 μm, respectively. However, the number of the droplets inside solid gains decreased evidently at 60 min and 90 min. Meanwhile, it can be found that some small liquid droplets gathered to form large droplets, and some of them penetrated the grain boundaries to form many irregular particles (marked with red ellipses) and small solid grains (marked with cyan round frames). As shown in Figure 3f, the solid particles were separated by the liquid phases and the size of the solid grains decreased to 153.2 μm. At 120 min, the shape factor of the solid grains increased to a relatively high value of 0.85, indicating that the spheroidization degree of solid grains was high. Besides, the distribution of liquid drops in the grain interiors was reduced obviously.

**Figure 4:**
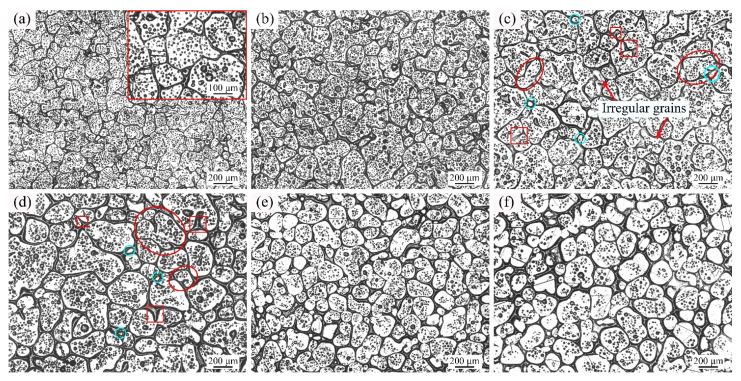
Optical micrographs of GH3536 alloy heat treated at 1360 °C for (**a**) 5 min, (**b**) 15 min, (**c**) 30 min, (**d**) 60 min, (**e**) 90 min, and (**f**) 120 min.

Figure 4 presents the optical micrographs of GH3536 alloy soaked at 1360 °C for various soaking times. Because the liquid fraction of GH3536 billets reached 40% at 1360 °C, liquid films were formed at the beginning of isothermal treatment. Therefore, the grain boundaries were relatively wide in Figure 4a. Even though, the intragranular droplets still occupied the majority of the liquid phases. At 5 min, the average size and shape factor of the solid grains were 135.0 μm and 0.77, respectively. In Figure 4b, the intragranular droplets grew up and more liquid phases occurred. As the heating time increased to 30 min and 45 min, the solid grains continued to grow, and their average sizes increased to 190.0 μm and 204.1 μm, respectively, as shown in Figure 4c,d. Meanwhile, due to the aggregation of liquid droplets (marked with red rectangular frames), some larger droplets destroyed the initial grain boundaries to form the irregular solid particles (marked with red ellipses). Meanwhile, some new grains with small sizes were formed (marked with cyan round frames). As shown in Figure 4e, at 90 min, the irregular solid grains were broken into some small spherical grains, so the mean size of solid grains decreased to 160.7 μm. As shown in Figure 4f, the mean size (147.3 μm) of solid grains at 120 min was smaller than that at 90 min, and there were more liquid phases between the solid grains.

**Figure 5:**
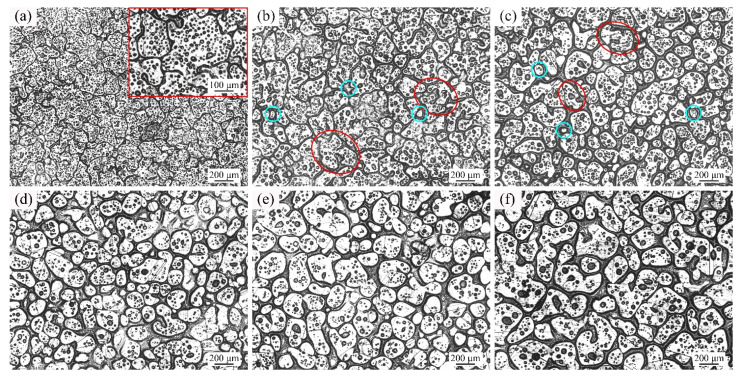
Optical micrographs of GH3536 alloy heat treated at 1364 °C for (**a**) 5 min, (**b**) 15 min, (**c**) 30 min, (**d**) 60 min, (**e**) 90 min, and (**f**) 120 min.

Figure 5 shows the optical micrographs of GH3536 alloy heat treated at 1364 °C for various heating times. As shown in Figure 5a, the solid grains with the average shape factor of 0.68 had irregular shapes and there were many small intragranular liquid drops. As shown in Figure 5b,c, the droplets grew up obviously and more liquid phases appeared. As shown in Figure 5d,e, the mean shape factors of the solid grains were increased to 0.84 and 0.83, respectively, and the number of liquid droplets in the solid grains was also significantly reduced. As the heating time increased to 120 min, the solid grains became larger (average size of 211.1 μm) and the shape of them became irregular (shape factor of 0.77), as shown in Figure 5f.

**Figure 6:**
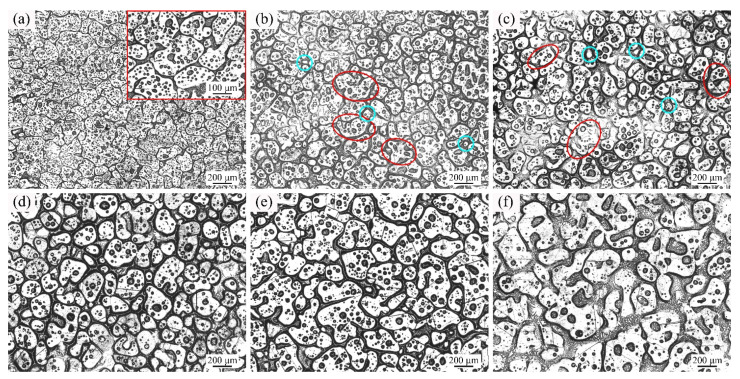
Optical micrographs of GH3536 alloy heat treated at 1367 °C for (**a**) 5 min, (**b**) 15 min, (**c**) 30 min, (**d**) 60 min, (**e**) 90 min, and (**f**) 120 min.

Figure 6 shows the optical micrographs of GH3536 alloy soaked at 1367 °C for various soaking times. As shown in Figure 6a, the microstructure of GH3536 alloy soaked at 1367 °C for 5 min presented similar characteristics compared to that at other temperatures. As shown in Figure 6b,c, at 15 min and 30 min, the proportion of liquid phases increased, and most of the grains obtained irregular shapes and there were some tiny solid grains with spherical shapes. As shown in Figure 6d, the solid grains grew obviously at 60 min, and their mean size was 148.0 μm. As shown in Figure 6e, when the heating time continued to increase to 90 min, the average grain size of 191.7 μm also increased. Meanwhile, it can be found that the liquid drops presented a trend of growth while the quantity of them was reduced greatly at longer heating times. As shown in Figure 6f, the solid particles had large mean size of 247.8 μm and small shape factor of 0.67. Meanwhile, the number of liquid droplets inside the grains was small while the sizes of them were large, and the liquid phases mainly distributed in the gap between solid particles at 120 min.

**Figure 7:**
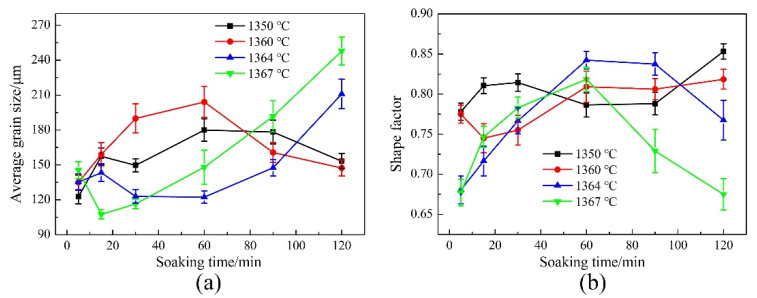
Variations of (**a**) average grain size and (**b**) shape factor of GH3536 alloy heat treated with different parameters.

Figure 7 shows the variations of the average grain size and shape factor of GH3536 alloy soaked for 5–120 min at various temperatures. As shown in Figure 7a, at 1350 °C and 1360 °C, the average grain size presented an increasing tendency before 60 min. From 60 min to 120 min, the average grain sizes decreased obviously at these two temperatures. At 1364 °C, the average grain size increased from 5 min to 15 min, then decreased from 15 min to 30 min. From 30 min to 120 min, it increased gradually. At 1367 °C, the average grain size presented a downward tendency from 5 min to 15 min, then it increased from 15 min to 120 min. The variation of shape factor in Figure 7b was closely related to that of the average grain size. At the beginning of heat treatment, the values of shape factors were all low. At 1350 °C and 1360 °C, the shape factor presented an irregular trend with the increase of time. However, at 1364 °C and 1367 °C, the shape factor increased first and then decreased, especially at 120 min, the shape factors were very low at these two temperatures.

### 3.3. Distribution of Elements in the Microstructure

**Figure 8:**
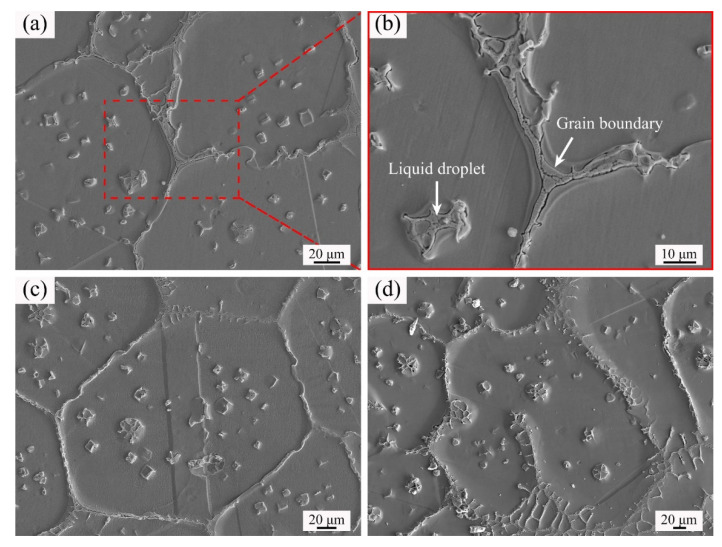
SEM images of GH3536 alloy after soaked for 60 min at various temperatures: (**a**) 1350 °C, (**b**) the enlarged image of (**a**), (**c**) 1364 °C, and (**d**) 1367 °C.

Figure 8 illustrates the SEM images of GH3536 billets soaked for 60 min at various temperatures. In Figure 8a,c, the grains exhibited equiaxed morphologies, meanwhile the small liquid drops inside the grains were easy to distinguish from the matrix. In Figure 8d, it can be found that the solid grains displayed irregular shapes and several liquid drops connected with the grain boundaries, which indicated the existence of breaking up mechanism. Figure 8b is the enlarged image of the region labeled in Figure 8a. It can be observed that there were some continuous precipitates on the grain boundaries. In addition, some precipitates were also found in the intragranular liquid droplets.

**Figure 9:**
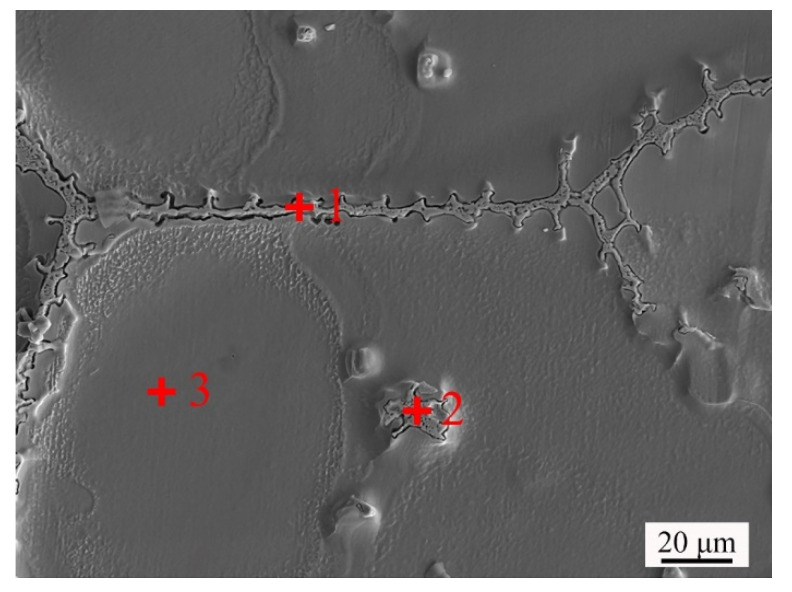
SEM image of GH3536 billet soaked at 1360 °C for 120 min.

Figure 9 is the SEM image of GH3536 billet soaked at 1360 °C for 120 min. The precipitates occurred on the grain boundaries (Location 1) and in the liquid drops (Location 2). The energy dispersive spectrometer (EDS) results of different locations in Figure 9 are listed in Table 1. It can be found that the chemical compositions of the precipitates of location 1 and location 2 were similar. Compared with the matrix (Location 3), the precipitates were rich in Cr, Mo, and Nb.

**Figure 10:**
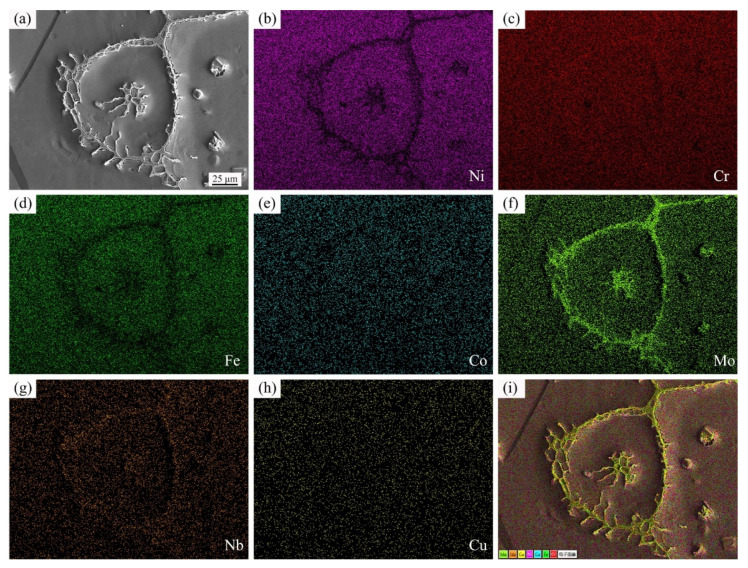
Map scanning of GH3536 alloy soaked at 1367 °C for 60 min: (**a**) SEM image, (**b**) Ni, (**c**) Cr, (**d**) Fe, (**e**) Co, (**f**) Mo, (**g**) Nb, (**h**) Cu, and (**i**) all alloying elements.

In order to identity the distribution of main elements of GH3536 billet after semi-solid isothermal treatment, the map scanning analysis was conducted. Figure 10 shows the map scanning results of GH3536 billet heat treated at 1367 °C for 60 min. Figure 10a presents the morphology of a complete solid grain, and the grain boundaries and liquid droplets can be obviously observed. According to the map scanning results in Figure 10b–i, it indicated that the contents of Cr, Mo, and Nb were more on the grain boundaries and liquid droplets, which was consistent with the EDS results in Table 1.

## 4. Discussion

In this work, the microstructure evolution of GH3536 semi-solid billets was affected by the isothermal temperature and soaking time. The average grain size and shape factor of solid grains were significantly different under different heating parameters, which was mainly influenced by the coarsening mechanism and refinement mechanism. As mentioned before, there are two main mechanisms for the coarsening of grains: the coalescence mechanism and the Ostwald ripening mechanism [29,30]. When the coalescence mechanism occurs, the solid grains need to contact with each other, so the proportion of liquid phase in the microstructure should not be too high [31]. As for Ostwald ripening mechanism, because of the different sizes of solid grains, the concentration gradient of the solid–liquid interface is different, leading to the melting of small grains and the growth of large grains [32]. The driving force of both the coarsening mechanisms is to decrease the total area of the interface and achieve the effect of reducing the total interface energy. Therefore, in order to keep the system stable, the solid grains tend to be larger and rounder. Besides the effect of grain growth mechanism, the semi-solid microstructure evolution is also influenced by the breaking up mechanism, which is mainly affected by the intragranular droplets [20]. Large intragranular droplets tend to break the grain boundaries and connect with the grain boundary liquid films, resulting in the formation of vermiculate grains. Hence, the breaking up mechanism will decrease the average size and shape factor of solid grains.

At 1350 °C, the liquid phase of 20% was relatively low and the solid particles were in close contact for short soaking times, so the coalescence mechanism worked (as shown the blue ellipses in Figure 3). Hence, the mean size and shape factor at 15 min and 30 min were larger than those at 5 min. When the soaking time was long, some liquid droplets would aggregate and break the grain boundaries (see the red ellipses in Figure 3), so the breaking up mechanism worked. Therefore, at 60 min and 90 min, some irregular grains were formed and the average shape factors were relatively low. At 120 min, the effect of breaking up mechanism was greater than that of coarsening mechanism, and many small globular grains were formed, which led to a decrease in grain size.

With the increase of temperature, the liquid fraction in the microstructure increased. The solid grains were separated by the liquid films and difficult to contact each other, which limited the effect of coalescence mechanism. Therefore, the dominant mechanism of grain growth was the Ostwald ripening mechanism at 1360 °C, 1364 °C, and 1367 °C. As shown in Figure 4, Figure 5 and Figure 6, with the increase of soaking time, many small liquid droplets aggregated and formed large drops, and some of them broke the grain boundaries to form vermiculate grains (see the red ellipses) and tiny spherical grains (see the cyan round frames). It should be noted that when GH3536 billets were heated for 120 min at 1364 °C and 1367 °C, the solid grains in the microstructure had irregular shapes and large sizes. Due to the high temperature and long soaking time, the effect of grain refinement and spheroidization was poor. It indicated that the semi-solid isothermal treatment needed to be conducted at a suitable temperature and time interval. In this work, the optimum parameters for preparing semi-solid billets of GH3536 alloy were: temperature of 1364–1367 °C and soaking time of 60–90 min.

Meng et al. [25] pointed out that the incipient melting occurred in the regions with a low melting point during the semi-solid isothermal treatment. In the process of heating, component segregation occurred on the grain boundaries and in the grain interiors, leading to the enrichment of a large number of alloying elements at these regions. As the result of incipient melting, the grain boundary liquid films and intragranular liquid droplets were formed [33,34]. After rapid quenching, these liquid phases were transformed into eutectic components. The EDS results in Table 1 and Figure 10 revealed that the eutectic components of GH3536 semi-solid billets were rich in Cr, Mo, and Nb, which proved that element segregation occurred in the liquid phases.

## 5. Conclusions

In order to verify the feasibility of semi-solid billets of GH3536 alloy prepared by SSITWS method, extruded GH3536 samples were directly heated to semi-solid temperatures and soaked for 5–120 min. The microstructure evolution during isothermal treatment was studied and the precipitates were characterized. The main conclusions were as follows:

- The microstructure of GH3536 semi-solid billets consisted of solid particles and liquid phases. The liquid phases were composed of intragranular liquid droplets and grain boundary liquid films.
- The heating temperature and soaking time had significant effects on the microstructure of GH3536 billets. The optimum parameters for fabricating GH3536 semi-solid billets were temperature of 1364–1367 °C and soaking time of 60–90 min. At these conditions, the solid grains had spherical shapes and suitable grain sizes.
- The microstructure evolution of GH3536 alloy was affected by the coalescence mechanism, Ostwald ripening mechanism and breaking up mechanism. The coalescence mechanism was dominant at short soaking times and low liquid fractions, while the Ostwald ripening mechanism worked at long heating times and high liquid fractions. When the intragranular liquid droplets were large enough, the breaking up mechanism occurred, resulting in the formation of vermiculate grains.
- The SEM observations and EDS results indicated that the eutectic components rich in Cr, Mo, and Nb were transformed from the grain boundary liquid films and intragranular liquid droplets after rapid quenching.

## Figures and Tables

**Table 1:** EDS results of different locations marked in Figure 9.

Location	-	Cr	Fe	Co	Ni	Cu	Nb	Mo
1	wt %	30.35	10.59	0.70	20.37	0.25	0.26	37.48
	at %	38.16	12.39	0.78	22.69	0.26	0.18	25.54
2	wt %	32.35	10.24	0.57	18.22	0.11	0.84	37.67
	at %	40.70	12.00	0.63	20.28	0.12	0.59	25.68
3	wt %	21.68	20.13	0.75	49.56	0.00	0.11	7.77
	at %	24.29	20.99	0.74	49.18	0.00	0.07	4.73

## Data Availability

The data that support the findings of this study are available from the corresponding author upon reasonable request.
